# Crosstalk of Hyperglycaemia and Cellular Mechanisms in the Pathogenesis of Diabetic Kidney Disease

**DOI:** 10.3390/ijms252010882

**Published:** 2024-10-10

**Authors:** Esienanwan Esien Efiong, Homa Bazireh, Markéta Fuchs, Peter Uchenna Amadi, Emmanuel Effa, Sapna Sharma, Christoph Schmaderer

**Affiliations:** 1 Research Unit of Molecular Epidemiology, Helmholtz Zentrum München, 85764 Neuherberg, Germany; 2 Institute of Epidemiology, Helmholtz Zentrum München, 85764 Neuherberg, Germany; 3 Department of Biochemistry, Faculty of Science, Federal University of Lafia, PMB 146, Lafia 950101, Nigeria; 4 Faculty of Medicine, Ludwig-Maximilians-University München, 81377 München, Germany; 5 Department of Pediatrics, Faculty of Medicine and Dentistry, University of Alberta, Edmonton, AB T6G 2R3, Canada; 6 Department of Biochemistry, Imo State University, Owerri 460222, Nigeria; 7 Division of Nephrology, Department of Internal Medicine, Faculty of Clinical Sciences, University of Calabar, PMB 1115, Calabar 540271, Nigeria; 8 German Research Center for Environmental Health, Helmholtz Zentrum München, 85764 Neuherberg, Germany; 9 Abteilung für Nephrologie, Klinikum Rechts der Isar, der Technischen Universität München, 81675 München, Germany

**Keywords:** advanced glycation end-products, autophagy, diabetes mellitus, diabetic nephropathy, end-stage renal disease, insulin resistance, microalbuminuria, podocytes, renal fibrosis, renin-angiotensin-aldosterone system

## Abstract

Among all nephropathies, diabetic kidney disease (DKD) is the most common cause of kidney impairment advancement to end-stage renal disease (ESRD). Although DKD has no cure, the disease is commonly managed by strict control of blood glucose and blood pressure, and in most of these cases, kidney function often deteriorates, resulting in dialysis, kidney replacement therapy, and high mortality. The difficulties in finding a cure for DKD are mainly due to a poor understanding of the underpinning complex cellular mechanisms that could be identified as druggable targets for the treatment of this disease. The review is thus aimed at giving insight into the interconnection between chronic hyperglycaemia and cellular mechanistic perturbations of nephropathy in diabetes. A comprehensive literature review of observational studies on DKD published within the past ten years, with 57 percent published within the past three years was carried out. The article search focused on original research studies and reviews published in English. The articles were explored using Google Scholar, Medline, Web of Science, and PubMed databases based on keywords, titles, and abstracts related to the topic. This article provides a detailed relationship between hyperglycaemia, oxidative stress, and various cellular mechanisms that underlie the onset and progression of the disease. Moreover, it also shows how these mechanisms affect organelle dysfunction, resulting in fibrosis and podocyte impairment. The advances in understanding the complexity of DKD mechanisms discussed in this review will expedite opportunities to develop new interventions for treating the disease.

## 1. Introduction

Diabetes mellitus is a metabolic condition marked by abnormal glucose homeostasis and is usually characterised by persistent hyperglycaemia, owing to defects in insulin secretion by pancreatic beta cells, lower sensitivity of cell surface receptors to insulin, or both [1,2,3]. 

Type 1 and type 2 diabetes mellitus cause severe dysregulation of glucose metabolism [4], resulting in hyperglycaemia. Chronic hyperglycaemia mediates mostly microvascular complications, including nephropathy, neuropathy, and retinopathy [5]. Of these, DKD has emerged as having one of the most devastating outcomes of the disease [3,6,7,8]. 

The kidneys play important roles in human physiology [9]; hence, any compromise of the renal tissue can result in various severe diseases. The consequences are then hypertension, cardiac failure, and end-stage renal disease (ESRD), with the need for kidney replacement therapy (KRT). The occurrence of ESRD becomes evident when DKD gives rise to kidney failure caused by hyperfiltration, elevated albumin excretion, microalbuminuria, nodular and widespread glomerulosclerosis, and ultimately, proteinuria [10,11,12]. Diabetic kidney disease is a difficult clinical challenge because of its common occurrence and insidious manner in its early stages [12], resulting in increased morbidity and mortality worldwide [6,13]. 

The outcomes for people with diabetes who develop DKD are significantly worse compared to those without the disease [14]. It is estimated that one in three individuals with diabetes develops nephropathy [15]. The prevalence of DKD varies across different populations and is strongly associated with the duration and severity of diabetes. As diabetes continues to surge worldwide, the burden of DKD is also escalating, adding considerably to the overall burden of CKD [16]. Unfortunately, there is no cure for the disease. The difficulty in finding a cure for DKD is mainly due to a poor understanding of the underpinning complex cellular mechanisms that could be identified as druggable targets for the holistic and precise treatment of DKD.

In this article, the objective is to give an update on the interconnection of hyperglycaemia and the network of cellular perturbations underlying the onset and progression of DKD and their effect on the morphology and function of renal organelles. 

## 2. Discussion

### 2.1. Hyperglycaemia, Onset and Progression of Diabetes Kidney Disease, and Its Diagnosis

Diabetic kidney disease begins with microalbuminuria [urinary albumin excretion (UAE) < 300 mg per 24 h], followed by macroalbuminuria (UAE > 300 mg per 24 h) [17], and then gradual loss of kidney function [18]. Persistent albuminuria in patients with DKD indicates glomerular assault. It may also suggest systemic endothelial dysfunction and extensive vascular injury [19]. 

The disease is diagnosed based on observed pathology of histological features [20], which can be divided into four classes: “thickened glomerular basement membrane (GBM), mesangial expansion, nodular sclerosis (Kimmelstiel--Wilson lesion), and severe glomerulosclerosis”, respectively. Along with these typical glomerular characteristics, interstitial fibrosis, interstitial fibrosis tubular atrophy (IFTA), arteriolar hyalinosis, and arteriosclerosis are also often found [14]. Changes in microalbuminuria, proteinuria, and creatinine clearance are helpful clinical markers of renal damage and therapeutic responses.

The advancement of DKD is typically characterised by glomerular hyperfiltration, microproteinuria, macroproteinuria, and a decreased glomerular filtration rate (GFR). Urinary protein (UP) concentrations [urinary albumin excretion rate (UAER)] for individuals with microproteinuria or 24-h UP and macroproteinuria are frequently used as key markers for DKD identification in clinical settings. Other markers may include renal function tests, blood glucose, cholesterol levels, and general symptoms [21]. 

Morphological and functional alterations in renal tissue comprise reduced podocyte function and its separation from the GBM. The ectopic buildup of extracellular matrix materials such as collagen type IV and VI, fibronectin, and laminin results in thicker GBM and mesangial matrix expansion. This is linked to tubule-interstitial fibrosis and glomerular sclerosis, which degrade kidney function and cause albuminuria and impaired GFR [22,23]. Chronic albuminuria and low GFR produce high blood pressure, which exacerbates DKD.

### 2.2. Interplay of Hyperglycaemia, Hyperinsulinaemia, Insulin Resistance (IR), and Diabetic Kidney Disease

Physiologically, when insulin binds to its receptor, it activates insulin receptor/insulin receptor substrate and insulin receptor/insulin receptor/phosphatidylinositol 3 kinases signalling cascades [24]. Glucose transporter (GLUT) type 4, the downstream signal for insulin receptor/insulin receptor/phosphatidylinositol 3 kinases/GLUT signalling, is mainly located in intracellular vesicles when there is no insulin stimulation. The activation of the insulin receptor initiates a cascade of cellular events that results in the translocation of GLUT4 to the extracellular membrane. This promotes the activity of GLUT4, leading to higher glucose absorption. Overexpression of GLUT1 causes higher extracellular glucose concentrations, decreased peripheral tissue glucose intake, glucose metabolic disruption, and high blood glucose levels [21].

Hyperglycaemia distorts the insulin/insulin receptor/phosphatidylinositol 3 kinases/GLUT signalling pathway, which results in hyperinsulinaemia and the activation of many inflammatory factors, such as cytokines and adhesion factors. These factors actively contribute to the occurrence of insulin resistance (IR) and diabetes complications, including DKD [25]. 

**Figure 1:**
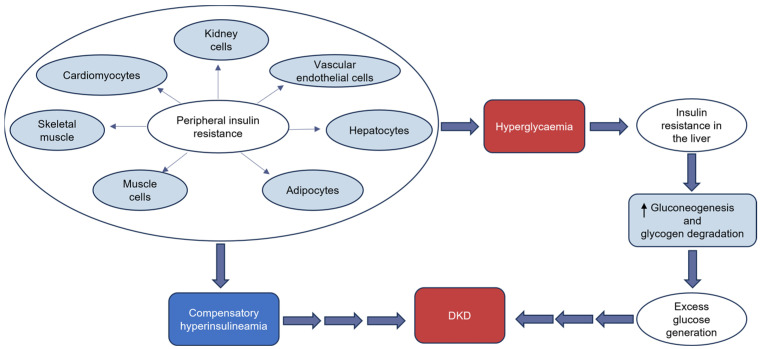
Interplay of hyperglycaemia, hyperinsulinaemia, insulin resistance, and diabetic kidney disease (DKD).

Insulin resistance is associated with the onset and aggravation of renal disease and is one of the factors that accounts for increased mortality in DKD [26]. Physiologically, in response to insulin, podocytes take up glucose via GLUT 1 and GLUT 4. However, hyperglycaemia causes podocytes to become insulin resistant [27]. This resistance is one of the primary causes of podocyte destruction in DKD [24]. Intracellular glucose metabolism disorders are recognised as being important for the pathogenesis of DKD [1,25,26,28]. Figure 1 shows the interplay of hyperglycaemia, hyperinsulinaemia, insulin resistance, and DKD. In Figure 1, peripheral insulin resistance leads to compensatory hyperinsulineamia and hyperglycaemia. High glucose levels cause IR in the liver, which increases gluconeogenesis and glycogen degradation, resulting in the production of excess glucose. These events eventually culminate in DKD.

### 2.3. Chronic Hyperglycaemia and the Pathogenesis of Diabetic Kidney Disease

**Figure 2:**
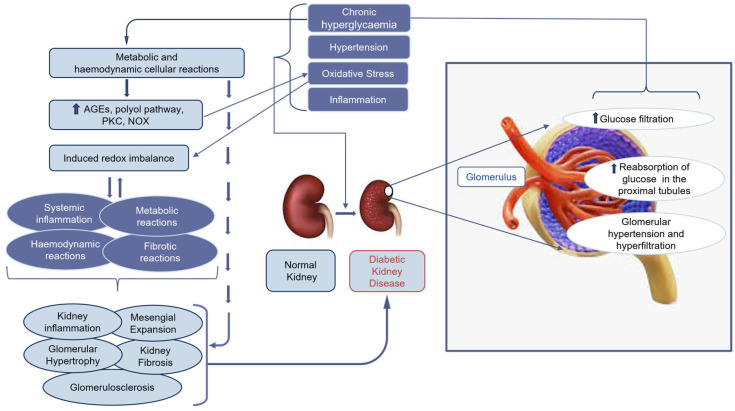
Contribution of the diabetic milieu to the progression of diabetic kidney disease (DKD). Advanced glycation end products (AGEs), nitric oxide (NOX), and protein kinase C (PKC).

The pathogenesis of DKD is complex and involves the crosstalk of different mechanisms [4,7,29], which originate from metabolic and haemodynamic cellular reactions that are activated by chronic hyperglycaemia. These cellular reactions give rise to advanced glycated end products (AGEs), protein kinase C (PKC), and nitric oxide (NOX) and feed the polyol pathway, causing the accumulation of reactive oxygen species (ROS). Thus, inducing redox imbalance gives rise to inflammation, fibrosis, metabolic, and haemodynamic reactions. These reactions result in activation of various pro-inflammatory pathways within the kidneys, mesangial expansion, thickening of the GBM, glomerulosclerosis, and renal fibrosis with subsequent development of DKD [6,23,30,31] (Figure 2). 

Hyperglycaemia, high blood pressure, OS, and inflammation are key to the development of DKD. The changes that occur following the development of DKD involve high glucose filtration, increased reabsorption of glucose in the proximal tubules, glomerular hypertension, and hyperfiltration [6].

### 2.4. Hyperglycaemia and the Contributors to Oxidative Stress in Diabetic Kidney Disease

#### 2.4.1. Oxidative Stress (OS)

Hyperglycaemia causes oxidative stress (OS) by triggering the synthesis of free radicals, which impairs the endogenous redox defence mechanism. An altered redox defence system promotes inflammation, autophagic dysfunction, fibrosis, and apoptosis by supporting MAPK/PKC/NF-κB/TGF-β1/mothers from decapentaplegic homolog (Smad) 2, 3, and 4/α-smooth muscle actin (α-SMA)/collagen signals] [32]. Elevated ROS is central to the pathogenesis of DKD [15] and is implicated in extracellular matrix (ECM) expansion and glomerular hyperfiltration [21]. 

Oxidative stress plays a key role in the first stage of DKD (Figure 2). It activates pathological pathways in the mesangial, epithelial, endothelial, podocytes, and tubular cells of the kidneys [6,23]. In diabetes, the primary producers of ROS are the polyol chain, AGEs, NADPH oxidases (NOX), and PKC (Figure 2). 

The isoform of the NADPH Oxidase (NOX)-NOX4 enzyme plays the most important role in the production of ROS in the kidneys [33]. Inhibiting the expression of NOX4 was shown to suppress ROS production [3]. The mechanisms of renal damage result in proteinuria and tubulointerstitial fibrosis [34]. This occurs because the glomerulus is more susceptible to oxidative damage than the rest of the nephron. 

The mechanisms of damage induced by OS can also take place indirectly by activating other pathogenic pathways to cause damage [35]. The sequence of events caused by OS collectively causes excess modification of the renal ECM in the mesangium. This enhances fibrotic processes in the tubular interstitium [15]. Podocytes are vulnerable to OS. Mature podocytes respond to injury by detaching from the GBM, autophagy, dedifferentiation, and apoptosis, thus causing proteinuria [36]. Vascular endothelial cells affected by hyperglycaemia have been shown to elevate OS and induce inflammation after blood glucose levels are normalised. Aberrant DNA methylation in mesangial cells has also been linked to the progression of DKD [18].

#### 2.4.2. Hyperglycaemia and Advanced Glycation End-Products (AGEs) 

**Figure 3:**
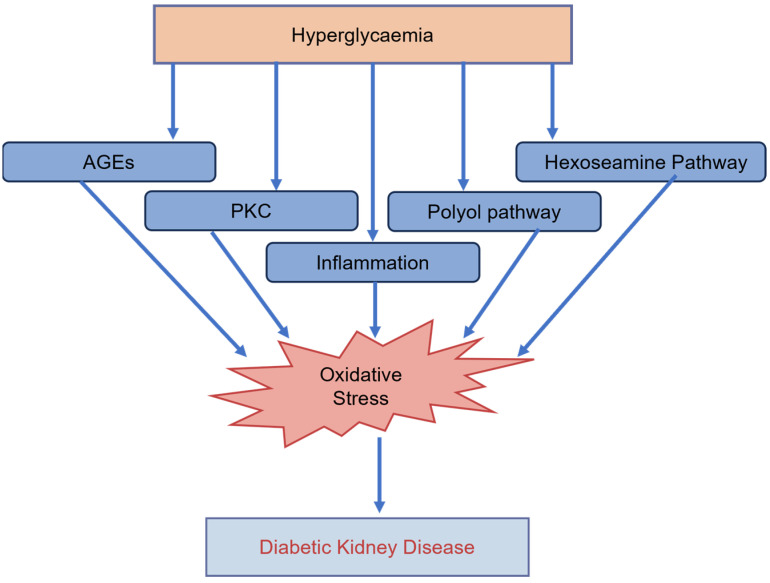
Hyperglycaemia and the cellular culprits in the generation of oxidative stress in diabetic kidney disease.

Chronic hyperglycaemia plays a critical role in the onset and aggravation of nephropathy [37,38] by contributing to intracellular and extracellular AGEs formation, [39] (Figure 3). 

AGEs are formed through non-enzymatic amino-carbonyl or Maillard reactions between the carbonyl groups of glucose, galactose, ribose, and fructose, or glucose metabolism intermediates (ribose-5-phosphate, glucose-6-phosphate, glyceraldehyde, fructose-6-phosphate, and deoxyribose-5-phosphate), with an amine group and other molecules to form a reversible Schiff base. This reaction eventually produces Amadori products, which are the initial products of the Maillard process [39]. 

Amadori products react strongly with amine groups and metal ions via glycoxidation of biological molecules, producing methylglyoxal, glyoxal, and malondialdehyde (MDA) [6,40]. A prolonged, elevated rate of glycolysis induces hyperactivation of the hexosamine and polyol metabolic pathways, increasing the formation of AGEs [6]. The expression of receptors of AGEs (RAGEs) and the accumulation of AGEs correlate with the pathogenesis of DKD [18].

#### 2.4.3. Hyperglycaemia and Inflammation

Prolonged low-grade inflammation is a pathophysiologic characteristic of diabetes [41,42]. Similarly, DKD is a low-grade chronic inflammatory disease [8] (Figure 3) because activation of inflammatory pathways is a crucial promoter of the initiation and development of the disease [42]. Local, persistent inflammatory stress damages organs, causes cell death, and affects the antioxidant defence system. This confirms the connection between inflammation and OS in the pathogenesis of DKD [42]. Activation of the renin-angiotensin-aldosterone system (RAAS) has been associated with a variety of inflammatory processes in the kidney [41,43]. 

Various other inflammatory factors are implicated in the renal response, which causes inflammation. For example, patients with DKD have higher expression of IL-1β in their serum and kidneys, which contributes to the disease’s development [44]. Patients with diabetes can have elevated levels of tumour necrosis factor-α (TNF-α) in their serum, leading to decreased GFR and vasoconstriction. Upregulation of monocyte chemoattractant protein-1 (MCP-1) and activation of the transcription factor NF-κB contribute to renal damage [21]. In DKD, the ongoing effects of inflammation culminate in the recruitment of fibroblasts, which eventually leads to renal fibrosis [44] (Figure 2). 

Proinflammatory cytokines, chemokines, cellular ligands, growth factors, leukocyte adhesion molecules [e.g., IL-6, IL-23, macrophage inflammatory protein 1β (MIP-1β), granulocyte monocyte-colony stimulating factor, and prostaglandin E2] [31], NO, inducible nitric oxide synthase (iNOS), ROS, and various receptors [45] also contribute to inflammation-related kidney injury in DKD. 

The accumulation of inflammatory macrophages in the kidney’s interstitium is linked to glomerulosclerosis, tubular atrophy, albuminuria, interstitial fibrosis, and renal function loss. Albuminuria causes an increase in protein uptake in the proximal tubules of nephrons, boosting the tubular protein burden and activating signalling cascades that produce inflammation and fibrosis in the tubular-interstitial area [45]. Thus, suppression of inflammation and glomerular endothelial cell dysfunction could be advantageous in the delay of DKD progression.

#### 2.4.4. Hyperglycaemia and Protein Kinase C (PKC) 

Hyperglycaemia activates metabolic pathways through PKC, a family of enzymes widely implicated in the progression of DKD [46]. Hyperglycaemia causes abnormal PKC activation (Figure 3), which increases intracellular AGEs, angiotensin (Ang II) formation, and augmented polyol and hexosamine pathway fluxes, which also play an important role in free radical generation [47]. 

Chronic hyperglycaemia causes abnormal activation of PKC by upregulating the conversion of glyceraldehyde-3-phosphate (GAP) to dihydroxyacetone phosphate (DHAP) and diacylglycerol (DAG), which results in sustained activation of PKC. This increases the production of connective tissue growth factor, vascular endothelial growth factor, TGF-β1, fibronectin, nitric oxide (NO), and collagen IV, which collectively play a critical role in the development of DKD [17].

#### 2.4.5. Hyperglycaemia, Hexokinase, and the Polyol Pathway

The polyol pathway is considered one of the main mechanisms responsible for the pathogenesis of diabetic complications. Excess glucose in the cells saturates hexokinase in the glycolytic pathway, thus shunting glucose metabolism toward the non-glycolytic mechanism called the polyol pathway [23] (Figure 3). The metabolism of glucose via the stimulated polyol pathway results in the accumulation of fructose and sorbitol in tissues. Imbalance in NADPH redox and changes in signal transduction also occur following activation of the polyol pathway [33]. Two enzyme systems are involved in this pathway. The first is aldose reductase, which is responsible for converting glucose to sorbitol using its cofactor NADPH. The second is sorbitol dehydrogenase, which converts sorbitol to fructose in the presence of the cofactor NAD^+^.

Activation of the polyol pathway results in a decreased concentration of intracellular NADPH, leading to increased ROS production and elevated cellular OS. This, in turn, reduces the activities of antioxidant enzymes such as superoxide dismutase and catalase, ultimately leading to progressive cell death. Studies on patients with stages 1 to 5 of CKD revealed that serum dimethylarginine (ADMA) and OS markers (erythrocyte SOD, GSH-Px, and plasma malondialdehyde (MDA)) were directly linked with different phases of CKD. In addition, GFR was adversely associated with plasma levels of ADMA and MDA but positively associated with erythrocyte GSH-Px and SOD levels [36]. Oxidative stress accounts for direct and indirect injury to mesangial, podocytes, and endothelial cells of the kidney [23]. 

### 2.5. Crosstalk of Hyperglycaemia, Conditions, and Mechanisms Underlying the Pathogenesis of Diabetic Kidney Disease

#### 2.5.1. Renin-Angiotensin-Aldosterone System (RAAS) 

**Figure 4:**
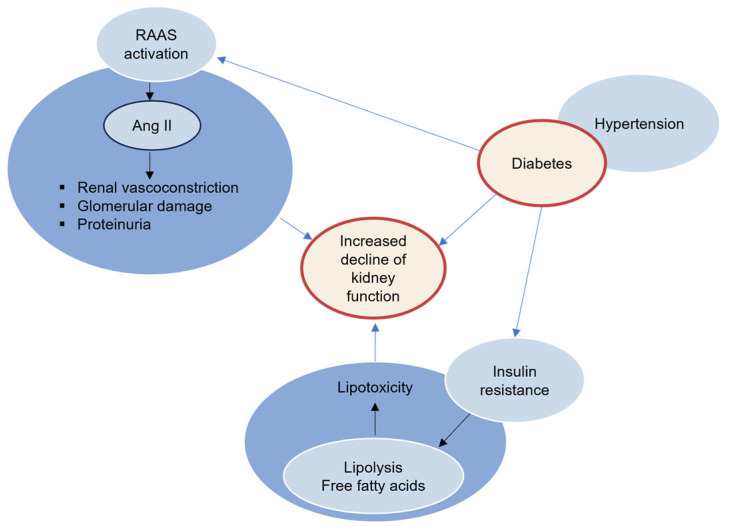
Crosstalk of diabetic milieu, RAAS, lipotoxicity, and hypertension to DKD.

In chronic hyperglycaemia, free glucose activates the renin-angiotensin-aldosterone system (RAAS) [3] (Figure 4). The angiotensin-converting enzyme (ACE; EC 3.4.15.1) is a key RAAS enzyme that converts the inactive protein angiotensin I (Ang I) to the active vasoconstrictor Ang II, raising blood pressure. It also induces the breakdown of bradykinin, which regulates vasodilation, natriuresis (the exclusion of sodium ions through urine), and OS. The enzyme is mostly found in pulmonary capillaries, as well as endothelial and epithelial cells in the kidney [48].

Activation of RAAS plays a significant role in DKD progression [23,49]. Its activation raises Ang II levels, which causes vasoconstriction of the efferent arteriole, resulting in increased glomerular hydrostatic pressure and hyperfiltration. The activation of RAAS induces aldosterone production, which causes renal vasoconstriction, glomerular damage, and proteinuria [17]. 

Inhibitors of RAAS, such as angiotensin-converting enzyme inhibitors (ACEIs) and Ang II receptor blockers (ARBs), drastically reduced the course of DKD [17,23,50,51,52], having success rates from 30% to 60% by lowering proteinuria and maintaining renal function [53]. In a similar way, inhibiting RAAS has been proven to be linked to long-term improvements in patient outcomes for a period of four decades [14].

#### 2.5.2. Hypertension

Hypertension often coexists with type 2 diabetes and affects about 70% of people with the disease. It is a significant risk factor for the initiation and development of DKD [36,38,54]. The combination of diabetes and high blood pressure creates a synergistic effect, causing additional stress on the delicate structures of the kidneys, which promotes the progression of renal injury [30,55] (Figure 4).

In the early stage of DKD, single nephron hyperfiltration and intraglomerular hypertension accelerate the decline in kidney function [52] (Figure 2). The combination of both conditions results in endoplasmic reticulum (ER) stress, impaired Ca^2+^ homeostasis, and mitochondrial dysfunction. These synergistically promote cellular apoptosis and contribute to the progression of DKD [55].

#### 2.5.3. Lipotoxicity 

Insulin resistance results in elevated lipolysis and high concentrations of free fatty acids (FFAs) in fat cells, which progresses in lipotoxicity [56]. Prolonged hyperglycaemia and hyperlipidaemia cause overproduction of free radicals, which induces OS and contributes to the reduction of vital β-cells [1,34,40], initiating the development of DKD [11] (Figure 4). 

The kidney biopsies of patients with DKD showed accumulation of lipid droplets in different renal cells when compared with healthy cells [57]. Dyslipidaemia significantly contributes to the aggravation of DKD by inducing a pro-inflammatory state and OS, which fosters the progression of renal injury [58]. 

#### 2.5.4. Podocyte Dysfunction 

Endothelial cells, podocytes, and GBM make up the selective glomerular filtration barrier (GFB) that inhibits loss of protein from the blood into the dominant filtrate [26]. Podocyte is a highly specialised and primary cellular component of the glomerulus, wrapping around capillaries and neighbouring cells of the Bowman’s capsule [24]. Podocytes cannot proliferate; hence, they are the most sensitive component of the GFB. 

**Figure 5:**
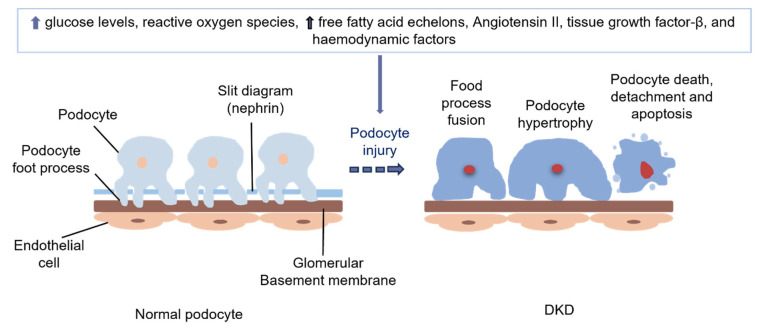
Pathophysiology underlying podocyte damage in diabetes.

Increased glucose levels, free radicals, Ang II, FFA echelons, TGF-β, and haemodynamic factors contribute to structural stress, causing changes in capillary tension and damage to the podocytes [26] (Figure 5). In both clinical and experimental DKD, proteinuria is associated with the loss of podocytes, one of the key cellular components of the GFB [23]. Thus, podocyte damage to the podocyte is the central link between the onset and development of DKD [59].

One of the main causes of podocyte injury in DKD is IR. To sustain podocyte viability and GFB integrity, insulin-stimulated podocytes activate the insulin receptor and the downstream PI3K/Akt cascade. Conversely, IR increases the risk of renal dysfunction in DKD [60]. In DKD, insulin signal transmission pathway errors of the podocyte give rise to many pathological processes [20,26]. Podocyte injury disrupts the filtration process, leading to proteinuria and the initiation of a cascade of events that contributes to kidney damage [61]. As DKD progresses, metabolic failure, OS, ER stress, and inflammation may result in irreversible podocyte destruction, aberrant apoptosis, and autophagy. 

In DKD, podocyte damage manifests as fusion, loss of foot processes, and apoptosis (Figure 3). The loss of podocyte integrity (cytoskeleton and slit diaphragm), or even its loss, is a critical cytological process that culminates in proteinuria, initially manifesting as microproteinuria and later macroproteinuria [60]. Therefore, the protection of the podocyte is important in the management of DKD.

#### 2.5.5. Autophagy Dysfunction 

Autophagy is an intracellular catabolic process that involves lysosome participation in the ageing and degradation of damaged organelles and proteins [62]. This lysosomal degradation pathway maintains cellular homeostasis by removing damaged organelles and preventing protein accumulation. Autophagy regulates the physiological and pathological processes of the kidney, and its activation is strongly linked to renal disorders, including DKD [59,63]. 

Inadequate autophagy contributes to the susceptibility of renal tubular cells, resulting in substantial tubular cell destruction and rapid disease development. Impaired autophagy may cause dysfunction of the mitochondria and higher ER stress in DKD [64]. Podocyte damage limits autophagy, resulting in the buildup of significant amounts of proteins (proteinuria) among people with diabetes and nephropathy [59].

#### 2.5.6. Epithelial-to-Mesenchymal Transition (EMT) 

One of the underlying causes of DKD is epithelial-to-mesenchymal transition, which involves the loss of epithelial cell markers like cadherins and the elevation of mesenchymal markers such as fibronectin and vimentin [65,66]. This represents the main pathological process that results in ESRD in several kidney diseases, including DKD [62,67,68,69]. The EMT process is induced by fibrogenic mediators such as TGF-β [65,70,71,72]. The transition causes the epithelial cells to lose their cubic shape [70], apical-basal polarity/junctions [62,63], and undergo cytoskeleton reorganisation [73]. Subsequently, these cells acquire phenotypes typical of mesenchymal cells, including a fibroblastoid-like appearance characterised by expression of α-SMA [68,70].

During EMT, epithelial cells fail to cross the GBM to form myofibroblasts. Instead, they assume mesenchymal features to release signals containing increased mesenchymal markers [74]. These mesenchymal cells are highly migratory and invasive, resulting in the buildup of ECM elements [75]. The change in morphology and phenotypic features that causes their elongation into spindle-shaped myofibroblasts occurs in four steps: the loss of epithelial cell adhesion, expression of fibrous characteristics, matrix metalloproteinase changes of tubular basement membrane, and enhancement of cell migration [65]. 

Fibroblast-specific protein 1 (FSP-1) referred to as α-SMA, S100A4, fibronectin, and collagen I is a reliable marker for characterising mesenchymal products produced by EMT throughout the development of fibrosis in various organs [72,75]. These mesenchymal cells contribute to wound healing, stem cell behaviour, and the progress of fibrosis and cancer [73]. By activating the Smad2/3 signalling process, renal tubular epithelial cells are driven towards EMT, which enhances ECM production and accumulation, ultimately leading to widespread renal tissue fibrosis [76].

### 2.6. Hyperglycaemia and Renal Organelle Dysfunction in Diabetic Kidney Disease

#### 2.6.1. Endoplasmic Reticulum (ER) Stress 

The ER is a well-orchestrated and complex cytosolic organelle that folds nascent polypeptide chains and traffics proteins. Inducers of ER stress (hyperglycaemia, FFAs, and lipoproteins) disrupt proteostasis, resulting in a buildup of misfolded/unfolded proteins in the ER lumen. This disrupts calcium homeostasis and triggers the unfolded protein response [77]. The underlying mechanisms of IR include ER stress, OS, inflammation, and mitochondrial dysfunction [78,79]. 

Endoplasmic reticulum stress has been identified as one of the pathogenic factors in DKD [79]. From the molecular scale, ER stress connects the inflammatory process with IR [26]. In T2D and DKD, markers of ER stress (inositol-requiring enzyme 1α, CCAAT-enhancer-binding proteins (C/EBP) homologous protein, activating transcription factor 6, glucose-regulated protein 78, protein kinase RNA-like ER kinase, OS markers, thioredoxin-interacting protein, and cytochrome b-245 light chain) alongside crosstalk markers (ER oxidase-1α and protein disulphide isomerase) are gradually elevated. This interconnectivity of crosstalk biomarkers suggests a positive connection between ER stress and OS markers [77]. 

Hyperglycaemia directly induces ER stress and apoptosis in podocytes [55]. As DKD progresses, ER stress, OS, metabolism dysfunction, and inflammation result in irreversible podocyte injury, abnormal apoptosis, autophagy, and disappearance of podocytes [21]. Renal ER stress is linked to the development of chronic renal failure and tubulointerstitial degeneration [26,80,81]. Non-mitochondrial ROS generation mechanisms include NADPH enzymes, inflammatory factors, and the endoplasmic reticulum [3]. 

The ER is attached to the mitochondria by mitochondria-associated membranes [82], which are made up of ER and outer mitochondrial membrane fragments. Oxidative and ER stressors are two closely connected physiological processes that interact and influence one another. In the initial phases of ER stress, calcium gets released from the ER, causing mitochondrial reuptake [55]. The increasing calcium concentration stimulates mitochondrial metabolic activity and ROS generation. This increases ROS through the action of cyclic-ADP ribose, which leads to enhanced ER calcium release. This could increase ROS generation, open the mitochondrial permeability transition pore, and cause mitochondrial destruction, creating a vicious cycle [3]. Advanced glycation end-products activate MAPK/ERK signalling, ER stress inflammation, OS, and fibrosis, and ultimately accelerate changes in the kidney pathology [83]. 

#### 2.6.2. Mitochondrial Dysfunction 

The kidney constitutes one of the most energy-demanding organs, with the second greatest expression of proteins related to oxygen consumption and mitochondrial function [81]. The kidney needs energy to reabsorb solutes, remove waste, maintain fluid, electrolytes, and acid-base balance. Therefore, the dysfunction of mitochondria contributes significantly to the pathophysiology of DKD and other renal conditions [57,84]. Several mitochondrial malfunctioning mechanisms have been identified as the leading molecular drivers of podocyte damage. They include reduced mitochondrial biogenesis and increased mitochondrial ROS generation [85]. 

Hyperglycaemia results in oxidative stress, which leads to organelle failure, particularly mitochondrial dysfunction, and ultimately energy failure [86]. Altered mitochondrial energetics in DKD induce changes in the electron transport chain, which increases ROS and decreases ATP production. This leads to increased mitochondrial divisions, decreased PGC1α levels, altered mitochondrial morphology, and elevated cell apoptosis, worsening DKD [82]. Protein filtering demands a lot of energy; hence, podocytes have a lot of mitochondria. Thus, podocyte mitochondrial dysfunction affects ATP production and causes OS and apoptosis, which accelerates the development of DKD [87]. Mitochondrial injury in glomerular endothelial cells and podocytes hence participates in the development of DKD [64,88]. 

## 3. Materials and Methods

Google Scholar, Medline, Web of Science, and PubMed databases were explored for relevant articles, which were published in English. The articles were based on observational studies on diabetes, CKD, DKD, and DKD mechanisms published within the last ten years, with 57 percent published within the past three years. The search was performed between December 2023 and July 2024. We used Boolean operators ‘AND’ and ‘OR’ and search terms such as DKD AND mechanisms, diabetic nephropathy AND mechanisms, chronic kidney disease in diabetes AND mechanisms, mechanisms underlying the onset AND progression of DKD, hyperglycaemia AND pathogenesis of DKD, end stage renal disease OR renal insufficiency, relationship between hyperglycaemia AND the mechanisms involved in DKD, high glucose milieu OR diabetic milieu, and DKD OR kidney replacement therapy in the title, abstract, and keywords of articles. Reports, letters to editors, and expert comments were excluded from the search.

## 4. Conclusions

This review aimed to understand the interplay between hyperglycaemia and cellular mechanisms underlying the pathogenesis of DKD. After the article searches, eighty-eight articles met the inclusion criteria and were included in the study.

This review highlights the contribution of hyperglycaemia to mechanisms and conditions that are interconnected in the pathogenesis of DKD. High diabetes milieu causes hyperinsulinaemia and insulin resistance in different renal cells. Renal damage results in microalbuminuria, which causes morphological and functional changes in kidney tissues through different cellular processes. 

The review reported the synergy between hyperglycaemia, oxidative stress, cellular dysregulation, and dysfunction of organelles such as the endoplasmic reticulum and the mitochondria. Ultimately, fibrogenic mediators activated through these mechanisms contribute to renal tissue fibrosis, autophagy, and finally DKD. Understanding the cellular insults that underpin the pathogenesis of the disease and how they relate to fibrosis and autophagy has significant translational implications that will pave the way for a more holistic therapeutic intervention for the disease. 

These findings will connect bench to bedside by highlighting the importance of robust clinical studies for assessing the efficacy and safety of new interventions targeted simultaneously at RAAS, inflammatory cytokines, fibrotic targets, apoptosis signal mechanisms, etc. Integrating the knowledge of these molecular insights into clinical practice will result in personalised medicine and biomarker identification for improved prognostic accuracy, early detection, and monitoring of DKD progression.

Although the study is limited by the non-inclusion of signalling pathways, future research directions in DKD therapeutics should be multifaceted and targeted at the underlying mechanisms. There is also a need to validate preclinical findings targeted at mechanisms of DKD in various patient cohorts. This will ensure that therapeutic findings are broadly applicable. Furthermore, there is a need for more collaboration between basic scientists, clinical professionals, and healthcare experts to enhance the conversion of discoveries into standard healthcare practices. 

Although the findings from this review can be generalised to different DKD cohorts, outcomes may vary depending on the presence of other underlying conditions. Nevertheless, therapeutics for CKD in diabetes should target the underlying mechanisms of the disease in a spontaneous, synergistic, and multifaceted manner, therefore creating more effective treatments that will ultimately improve patient outcomes and reduce the global burden of DKD.
